# Availability, accessibility, and quality of adolescent Sexual and Reproductive Health (SRH) services in urban health facilities of Rwanda: a survey among social and healthcare providers

**DOI:** 10.1186/s12913-020-05556-0

**Published:** 2020-07-29

**Authors:** Pacifique Ndayishimiye, Rosine Uwase, Isabelle Kubwimana, Jean de la Croix Niyonzima, Roseline Dzekem Dine, Jean Baptiste Nyandwi, Justin Ntokamunda Kadima

**Affiliations:** 1 Youth Service Organization (YSO), Musanze and Huye Districts, P. O box 511, Huye, Rwanda; 2 University of Rwanda, College of Medicine and Health Sciences, Kigali, Rwanda

**Keywords:** Availability, Accessibility, Sexual and reproductive health services, Adolescents, Health providers, Urban

## Abstract

**Background:**

Adolescents are still getting pregnant and contracting Human Immunodeficiency Virus (HIV) and Sexually Transmitted Infections (STIs) in Rwanda as elsewhere. Quality and comprehensive SRH services and information for adolescents is valuable for adolescents’ wellbeing. This study aimed at understanding SRH services providers’ viewpoints on accessibility, availability, and quality of SRH services provided to adolescents in selected cities of Rwanda.

**Method:**

The study was a descriptive cross-sectional survey conducted between May 2018 and May 2019 in six selected cities of Rwanda using a mixed-methods approach. A checklist was used to collect data from 159 conveniently selected SRH services providers. The survey tool was validated. SPSS version 20 was used to describe quantitative data and ATLAS TI version 5.2 was used to code and analyze the qualitative data thematically.

**Results:**

Qualitatively, health care providers reported that the availability of adolescent SRHS are satisfactory with access to accurate SRH information, contraceptive methods, prevention and management of STIs and HIV services, and counselling. However, the accessibility of some services remains limited. According to respondents, some products such as female condoms are less in demand and often expire before they can be distributed. One nurse clarified that they render services at a low price if an adolescent has insurance medical coverture. Religious leaders and family members may hinder adolescents from health-seeking behavior by promoting abstinence and discouraging use of protective means. Quantitatively, we found that 94.3% of health facilities provide information to adolescents on SRH services that were available and 51.6% affirmed delivering services at a low cost. Only 57.2% of respondents mentioned that adolescents are involved in designing the feedback mechanisms at their facilities.

**Conclusion:**

SRH services in Rwanda are available for the general population and are not specifically designed for adolescents. These SRH services seem to be fairly accessible to adolescents with insufficient quality as adolescents themselves do not get to be fully involved in service provision among other aspects of quality SRH as stated by the World Health Organization (WHO). Therefore, there is a need to improve the present quality of these services to meet adolescents’ needs in an urban setting.

## Background

Globally, there are nearly 1.2 billion adolescents aged 10 to 19 years old, representing about 16% of the world’s population [1]. About 789 per 100,000 adolescents suffered adverse maternal outcomes in 2015, and nearly 3000 adolescents die each day from preventable causes related to sexual reproductive health (SRH) [2]. The International Conference on Population and Development (ICPD) in Cairo 1994, urged governments to make reproductive health services available, accessible, acceptable and affordable to young people [2–4]. During this meeting, reproductive health needs of young people were discovered to be largely ignored by existing health facilities, educational segments and other social programs. Improving their health could improve economic prosperity in all sectors in communities [5, 6].

Sexual and reproductive health services (SRHS) ought to provide health information, education and counselling, provide of a range of safe and affordable contraceptive methods, quality obstetric and antenatal care for all pregnant girls, testing (pregnancy and HIV), prevention and management of STIs, conduct promotional activities, and encourage active participation of adolescents [5]. It is crucial to promote adolescents’ health and an essential step toward achieving Sustainable Development Goals (SDGs). The World Health Organization (WHO) has also introduced guidance to help governments and SRH services providers respond to the growing health needs of adolescents and have suggested other interventions like the operation of youth-friendly clubs [7].

Despite these efforts, adolescents from Sub-Saharan Africa (SSA) are still being affected by sexual and reproductive health problems [8]. Adolescents’ full access to SRH services and information essential for the promotion of their human rights are still lacking in many SSA countries [3] and this is due to numerous barriers which they face in accessing services even when they are present [9]. Barriers reportedly faced by adolescents include lack of youth-friendly and comprehensive SRH services at many health facilities, shortage of trained personnel, conducive environment for adolescents, shortage of information on the services provided, and provider attitudes that are not friendly to young people and adolescents [9, 10].

Inadequate SRH services provided to adolescents increase the risks of unwanted pregnancies, unsafe abortion, HIV, STIs, and mental health problems in adolescents [7]. The under-utilization of the service package also leads to adolescents, especially girls, getting inaccurate SRH information from their peers and uninformed laypeople [11]. Several factors expose adolescents to sexual and reproductive health problems such as taboos surrounding sex education, early marriage, norms and traditions, and lack of promotion of comprehensive knowledge of sexual and reproductive health by public campaigns/entities/government [10, 12].

In many African countries like Uganda, Nigeria, and Botswana, sexual and reproductive health services for adolescents were reported to be of low quality, citing inconvenient hours of operation, long waiting time, and cost of services for adolescents [4, 13, 14]. Most African countries do not have sufficient trained staff to provide and cater for the SRH needs of adolescents [11].

Like other SSA countries, adolescents in Rwanda still face challenges while seeking or trying to access SRH services. Some issues which young people come across while in quest of these services are limited availability of specialized trained health care providers capable of catering to adolescents’ health needs, cultural mores and myths, religious beliefs, and peer pressure [15]. The government of Rwanda has been putting mechanisms and policies in place such as the Isange One Stop Centers and youth friendly corners in schools and health facilities to educate young people about SRH since 2010 [16]. However, there is evidence that despite these government policies, there are increasingly unwanted teen pregnancies, risky sexual behaviors, lack of comprehensive knowledge of SRH, and rising HIV infection especially among female adolescents in urban areas such as Kigali, Rwanda [15, 17, 18].

The provision of youth-friendly sexual and reproductive health services by ensuring availability, accessibility, and quality are essential for adolescents to live healthy lives and thrive in their communities [19, 20]. Quantitative and qualitative data are crucial to communicate SRH service provision for adolescents in Rwanda, whereby the services provided to adolescents are not yet well documented. Improving the SRH needs of adolescents can be achieved by understanding the views of health care providers on SRH services provided to adolescents. Nevertheless, there is a gap in knowledge about provision of SRH services to adolescents in urban settings of Rwanda. Adolescents’ access to sexual reproductive health’s services play a valuable role in their overall SRH. Understanding their perspective is important to improve access to SRH services for adolescents. Therefore, this study aimed to understand the SRH services providers’ view on availability, accessibility, and quality of SRH services provision among adolescents in selected urban settings of Rwanda.

## Methods

### Study settings

**Fig. 1:**
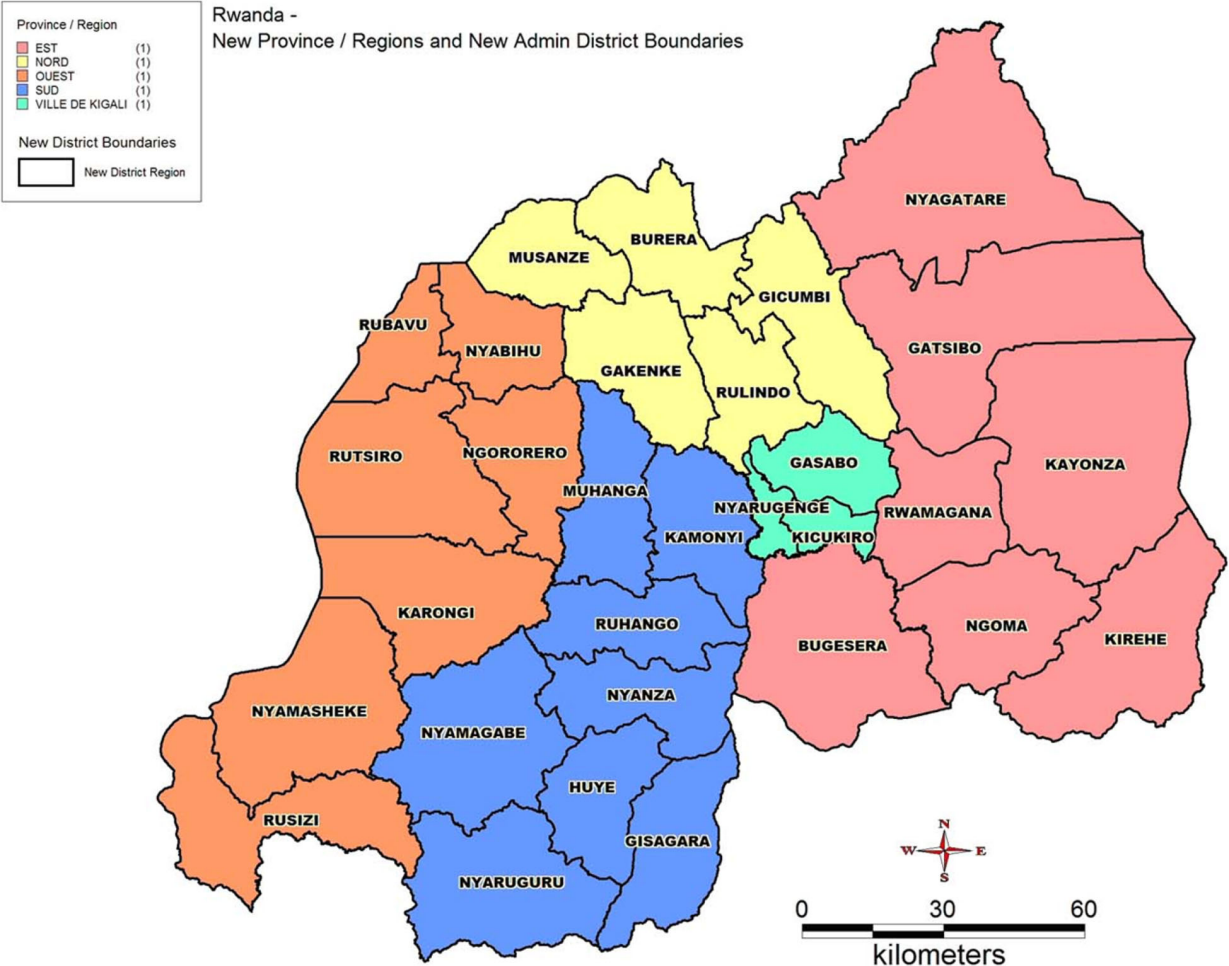
Administrative map of Rwanda (source: https://upload.wikimedia.org/wikipedia/commons/c/c4/Rwanda_Districts_Map.jpg)

Rwanda is structured into districts within the four provinces and Kigali city (Fig. 1). For this study, we surveyed six sites, including Kigali, Nyanza, Huye, Rwamagana, Musanze, and Rubavu, based on their population size and classification as cities in Rwanda. Each district has specialized youth-friendly health centers delivering Adolescent Sexual and Reproductive Health Services (ASRHS). The study took place in SRH services provision units or departments at the sites of data collection (health facilities).

This study was a prospective, descriptive, cross-sectional, mixed-methods survey conducted between May 2018 and May 2019 among social and healthcare providers in charge of ASRHS in the selected centers. The mixed-methods approach consisted of administering written semi-structured questionnaires to the respondents, followed by in-depth interviews.

### Study participants

The study enrolled 159 social and healthcare providers (54.5% men and 45.5% women), selected by convenience based on their responsibilities in the selected entities. Nurses, medical doctors, social workers and other SRH services providers at the study health facilities were interviewed. We recruited (41.3%) of study respondents from government facilities, 56.3% from private facilities, and (1.9%) from religious affiliated facilities. Most of them were recruited from Nyarugenge district health facilities (28.8%) and the least number came from Huye (5.6%), as shown in Table 1.

**Table 1:** Percentage of staff on SRHS per district

Workplace	
	**n (%)**
Musanze	13(8.1)
Huye	9(5.6)
Rubavu	16(10)
Rwamagana	16(10)
Gasabo	30(18.8)
Nyarugenge	46(28.8)
Kicukiro	28(17.5)
**Affiliation**	
Government	66(41.3)
Private	90(56.3)
Religious	3(1.9)

### Data collection

Health providers who agreed to participate answered both written and verbal questions. A checklist was adapted from the Pathfinder International (PI) checklist composed of questions designed to align with the World Health Organization (WHO)‘s expectations on the provision of Youth Friendly Health Services (YFHS) and Quality of Care Standards to accomplish the study objectives. The original checklist that was adapted for this study can be assessed as “*Pathfinder International (PI). Clinic Assessment of Youth Friendly Services*” and is referenced in this document as [21]. This checklist was designed with sections where respondents needed to respond YES (1) and No (0) or rate a question on a scale of five for the quantitative part. Each response from this quantitative section, had a follow up qualitative question to explain why the respondent answered in that manner. The lead researcher and three trained research assistants from Youth Service Organisation (YSO) and the University of Rwanda College of Medicines and Health Science (CMHS) were responsible for data collection. Research assistants were all fluent in Kinyarwanda and English. Interviews typically lasted an hour and a half to two hours.

### Study measures

To measure availability we looked at services available, availability of health information, continuity in service provision, available staff monitoring SRH services, available waiting rooms, and availability of equity when providing services to adolescents.

**Accessibility** was measured by; access in terms of location, via social media, accessibility in terms of appointment, cost of services, access in terms of frequency, access of medical record, and access age.

**Quality** was measured by using confidentiality and privacy in terms of entrance, service provision, accessibility, environment; written guidelines; staff characteristics and competencies; and adolescent involvement.

### Ethics

The study protocol obtained ethical clearance from the University of Rwanda, College of Medicine and Health Sciences Institutional Review Board, reference number CMHS/IRB/370/2018. Signed consent was obtained by interviewees before being interviewed.

### Data analysis

#### Quantitative data analysis

Data collected were cross-checked for completeness. The Statistical Package for the Social Sciences (SPSS) version 20.0 served for frequencies and descriptive statistics.

#### Qualitative data analysis

The availability of services corresponded to the physical presence of an item on an inventory list. Accessibility indicated the effective delivery of the service as impacted by different barriers. Quality measurements determined if adolescents were involved in SRH, management of confidentiality, and satisfaction of adolescents. Qualitative responses from the interviews recorded in Kinyarwanda were transcribed into English while maintaining the contexts of the responses. ATLAS TI version 5.2 was used to code and analyze thematic data. Logical reasoning and structured analytical techniques were employed to identify errors during data transcription, cleaning, and analysis.

## Results

### Socio demographic profile of study participants

**Fig. 2:**
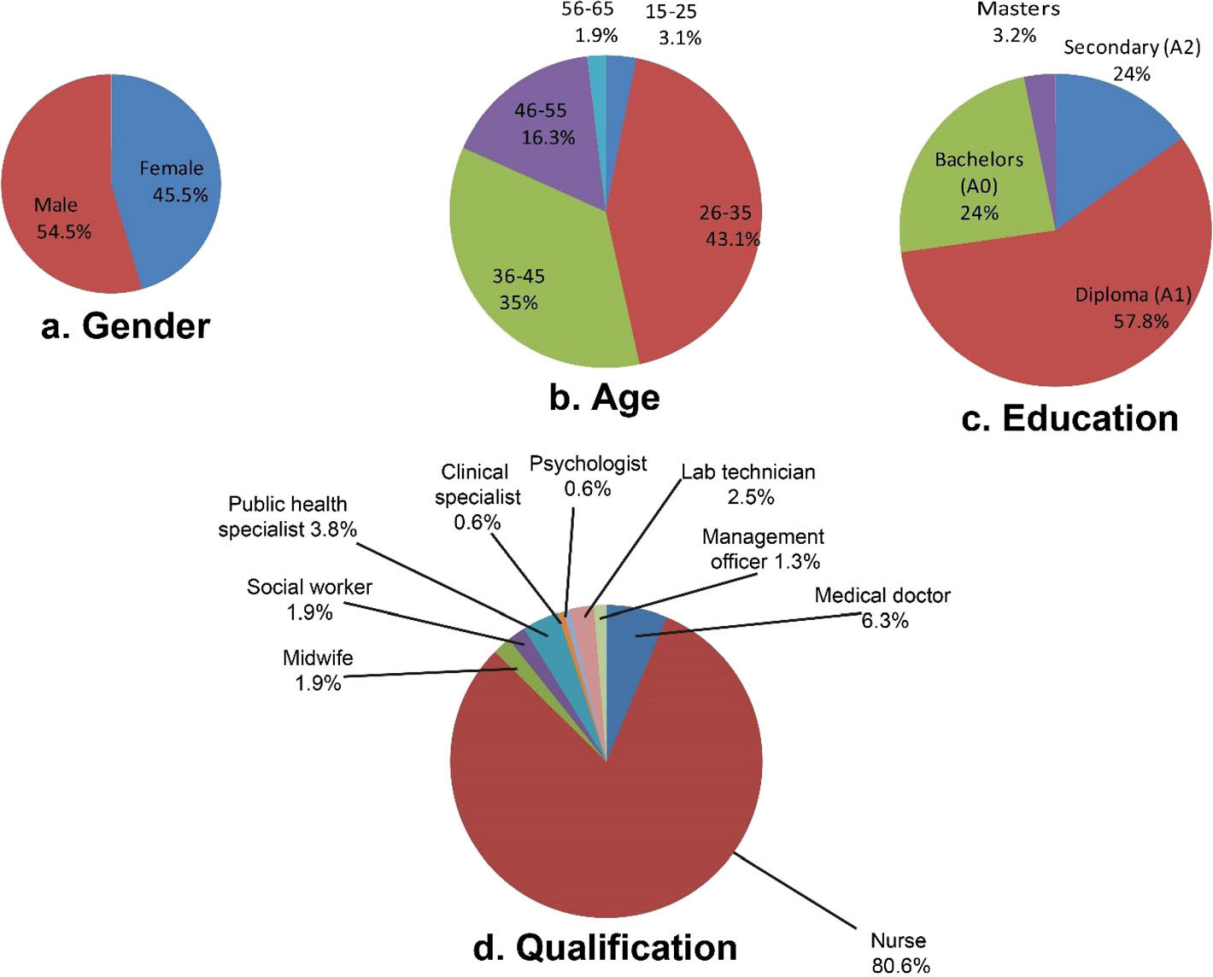
Social demographic characteristics of respondents

Figure 2, shows the socio demographic characteristics of the 159 respondents. There were 54.5% males and 45.5% females aged 15–65 years old with the majority (43.1%) aged between 26 and 35 years old.

Respondents held nine distinct job titles (Fig. 2 with a majority being nurses (80%) followed by general medical practitioners (6.3%). Education levels included secondary (14.9%), diploma (57.8%), bachelors (24%), and masters (3.2%).

### Availability of SRH services

Availability of services related to HIV and STDs themes at the surveyed health facilities are presented in Table 2. STDs testing was available in 92.3% of facilities, but only 64.8% of health facilities surveyed give treatment. HIV testing was available in 86.2% of facilities, but self-testing and self-testing kits were available in only 25 and 19.7% of the facilities, respectively. Only 42% of facilities give HIV treatment on the same day of diagnosis and only 31.6% facilities give treatment at any time. More than 80% of facilities offer HIV-counseling and 64.2% practice adult male circumcision. On the family planning related services, 85.5% of facilities may offer contraceptive methods, including combined oral pills (78.3%), progesterone pills (75.5%), male condoms (73.6%), female emergency contraceptives (63.9%), Depo-Provera injection (66%), hormonal implant (53.6%), intrauterine device (IUD) (49%), female condom (21%), lubricants (14.1%), vasectomy (11.5%), and tubal ligation (21.8%). Fertility awareness, antenatal counseling, and postnatal care may be available in 45–52% of facilities.

**Table 2:** HIV / family planning related SRHS Available at the surveyed health facilities

Variables	Total	YES n (%)	NO n (%)
STDs testing	159	145(91.2)	14(8.8)
STDs treatment	159	103(64.8)	56(35.2)
HIV testing	159	137(86.2)	22(13.8)
HIV self-testing	156	39(25)	117(75.0)
HIV Self-testing kits	157	31(19.7)	126(80.3)
HIV same-day therapy initiation	157	66(42)	91(58)
HIV treatment	158	50(31.6)	108 (68.4)
HIV counselling	158	127(80.4)	31(19.6)
Circumcision	159	102(64.2)	57(35.8)
Contraceptives	159	136(85.5)	23(14.5)
Combined oral contraceptive	157	123(78.3)	34(21.7)
Progesterone contraceptive	155	117(75.5)	38(24.5)
Emergency contraception	158	101(63.9)	57(36.1)
Depo Provera injection	150	99(66)	61 (34.0)
Implant	153	82(53.6)	71(46.4)
Intrauterine Device (IUD)	155	76(49)	79(51)
Male condom	159	117(73.6)	42(26.4)
Female condoms	157	33(21)	124(79)
Lubricants	156	22(14.1)	134(85.9)
Vasectomy	157	18(11.5)	139(88.5)
Tubal ligation	156	34(21.8)	122(78.2)
Fertility awareness	158	82(51.9)	76(48.1)
Antenatal Care (ANC)	157	75(47.8)	85(52.2)
Post-natal care	157	70(44.6)	87(55.4)

SRH services that adolescents could use, were mentioned to be available but often under-utilized and expire before being utilised, leading to wastage of scarce health resources.

*“The male’s condoms are available, but there is stock-out of the female’s condoms. The previous females’ condoms expired because the clients did not request them. The females say that they do not use female condoms because of the difficulty with the insertion of the condom during sex. The participants also do not like using them because of the difficulties in using them during sexual intercourse” — n*urse _in Gasabo District*.*

We also aimed to document the package of SRH services available at health facilities, and the respondents expressed that some services are not available at their facilities, due to the predetermined SRH health care package for health facilities that are set by the Ministry of Health guidelines. The respondents feel that if they were allowed to provide some services in their health facilities, they would have had all the SRH services needed by the adolescents.

*“This private institution provides the tests and treatments of STIs such as Syphilis, Trichomoniasis, Candidiasis, Chancroid. It also provides HIV counseling. For HIV treatments, HIV patients are transferred to public institutions for ART. We also have some contraceptive methods, but people who need family planning are also transferred to the hospital or health center where the governmental institutions provide these services for free. Moreover, pregnant women in our institution are cared for, for example, on echography, ANC and PNC care, and partial health interventions are provided. The challenge at our facility is a lack of rights to deliver all the SRH services, but we need to create a strong partnership with the Ministry of Health for us to provide them.* Said a medical doctor at a private clinic in Kicukiro.

### Accessibility of SRH services to adolescents

The majority of the respondents expressed that their facilities have been providing more information on SRH services (94.3%) and general health (85.7%) after they have provided a specific SRH service to an adolescent. Although more than half of respondents (59.4%) reported that they provided more time for interaction with adolescents, only 46.9% of staff were trained to provide SRH services to adolescents, as shown in Table 3.

**Table 3:** Accessibility of SRH services to adolescents at the surveyed facilities

Variables	Total	YES (%) n (%)	NO (%) n (%)
More information availed on services provided	159	150(94.3)	9(5.7)
More information on general health	154	132(85.7)	22(14.3)
Time for interaction	155	92(59.4)	63(40.6)
Referral mechanism	159	123(77.4)	36(22.6)
Job description and responsibilities	147	69(46.9)	78(53.1)
Information spread on services (Campaigns/awareness)	158	98(62)	60(38.0)
Social media usage	155	31(20)	124(80)
Waiting time	156	93(59.6)	63(40.4)
Service cost	155	80(51.6)	75(48.4)
Suitable operational hours	157	123(78.3)	34(21.7)
Access to medical records	158	144(91.1)	14(8.9)
Age at which adolescents should access the service	96	48(50)	48 (50)
**Accessibility in terms of location from residence to facility**			
Accessibility to location (Less than 30 min)	156	98(62.8)	
Accessibility in location (30 min^−1^ h)	156	53(34)	
Accessibility in location (1 h–2 h)	156	5(3.2)	

In-depth interviews showed that interviewees were providing information on where adolescents could access SRH services and their location but not on a routine basis. SRH Services providers felt that all the required information by the adolescents on SRH services are made available during ongoing campaigns organized by different institutions.

*“Health providers spread awareness about the available friendly Adolescents’ SRH services when there is a campaign, because adolescents attend it”.* Nurse in Huye*.*

The interviewees also lamented being over-worked by other health care services while providing SRH services. They were scared that the lack of detailed information about their responsibilities at work affected the time spent providing such services to adolescents.

“We don’t have an *organogram at our facility because we get assigned any other responsibilities within other units or departments other than the SRH department … … …*” Nurse in Gasabo.

Table 3 shows that the service providers believe that only (62.8%) of the adolescents travel less than 30- min walk to reach a venue for accessing SRH services, while the use of social media for education and information provision on SRH were not commonly used (72.2%) by the health facilities. Over half of respondents (51.6%) feel that close to half of the services are provided at a low cost and 49.4% reported that the needs of the adolescents were not being met in their facilities. Furthermore, most of the study respondents responded that they do not provide SRH information to adolescents through social media platforms like Facebook, Whatsapp, and Twitter.

“*Social media are not used at the health center because our health center still has the barriers to having required equipment and resources needed for providing the education and information using the social media platforms … … … ..*” Midwife in Rubavu.

The respondents believed that it is only at public facilities that adolescents can access SRH services at an average cost. They further said that for those who had access to any medical insurance, access to services is at a price worth searching for them at private facilities.

*“The services are provided at low cost because only 15% of the service cost is paid by the client who have health insurance. The patient with no health insurance must pay 100% of the treatments”,* Nurse in Nyarugenge added. “*Our institution provides various services for private clients, that is why the services we provide, including SRH services for adolescents, are paid 100%, but we accept the health insurance when it is relevant”* Nurse in Rwamagana responded.

### Quality of SRH services provided to adolescents

Tables 4, demonstrate the perceived quality of SRH services by services providers provided to adolescents within the urban health facilities in Rwanda. Regarding privacy, 64.3% of health facilities did not separate entrance for adolescents to use while they visit the health facility to access SRH services but 88.5% of respondents expressed that health facilities have private rooms for consultations.

“*The providers give SRH services to adolescents in privacy and confidential manners. The socio-demographic and patients’ status are kept in privacy*.” Medical Doctor in Musanze.

**Table 4:** Quality of SRH service provided to adolescents

Variables	Total	Yes n (%)	No n (%)
Health care providers receive essential training	157	91(58)	66(42)
Use of written guideline	154	49(31.8)	105(68.2)
Continuous learning	158	116(73.4)	42(26.6)
Staff oriented to provide confidential AFS	158	123(77.8)	35(22.2)
Staff are non-judgmental, friendly, welcoming, good listeners	157	149(94.3)	8(5.1)
Staff demonstrate respect when interacting with adolescents	155	144(92.3)	11(7.1)
Discreet entrance	157	56(35.7)	101(64.3)
Privacy in services provision	156	148(94.9)	8(5.1)
Confidentiality in accessibility	156	98(62.8)	58(37.2)
Privacy of the rooms	157	139(88.5)	18(11.5)
Comfortable waiting area	158	127(80.4)	31(19.6)
Staff Supervision	156	62 (39.7)	94 (60.3)
Time for results provision	157	148(94.3)	9 (5.7)
Non-discrimination in terms of educational materials	157	64(40.8)	93 (59.2)
Non-discrimination in terms of service provision	158	107(67.7)	51 (32.3)
Adolescent involvement in feedback provision on provided services	159	86(54.1)	73(45.9)
Involvement in the availability of peer educators	159	53(33.3)	106(66.6)
Adolescent involvement in designing feedback mechanism	158	67(42.1)	91 (57.2)
Needs of Adolescents satisfied (meeting needs)	156	79(50.6)	77 (49.4)

Table 5 shows that 68.2% of respondents did not present to the interviewer any written guidelines used for SRH services provided to adolescents. Additionally, the respondents felt that only 58.0% had accessed some training on providing SRH services to adolescents, while 73% had continuous access to adolescent SRH education. They added, however, that in-service training and documentation are conducted to provide services to adolescents, whereby they said that they provide SRH services to adolescents without any prejudices and stigmatization and that room for improvement is needed.

**Table 5:** Barriers or obstacles to access services for adolescents

Barriers to SRHS	Total (N)	YES n (%)	NO n (%)
None serves as a barrier	157	8(5.1)	149(94.9)
Community as a barrier	157	71(45.2)	86(54.8)
Family as a barrier	157	107(68.2)	50(31.8)
Friends as a barrier	157	27(17.2)	130(82.8)
SRHS staff as a barrier	157	23(14.6)	134(85.4)
Adolescent peers as a barrier	157	16(10.2)	141(89.8)
Policymakers as a barrier	157	16(10.2)	141(89.8)
Religious leader as a barrier	157	102(65)	55(35)

A total of 45.9% respondents said that they do not have formal mechanisms to receive feedback from the adolescents on services provided, and only 42.1% of respondents acknowledged to involve adolescents in designing the feedback mechanisms, while only 33.3% of the facilities make use of the adolescents peer educators in SRH services and information provision to adolescents. Respondents expressed respect for privacy and confidentiality while providing SRH services to adolescents. Only half of adolescents have their needs completely satisfied, resulting in half of visits encountering at least one obstacle in accessing the services available.

*“The clinic does not have a trained health provider about the SRH services and there is no specific health provider for adolescents seeking SRH services”* Nurse in Rubavu. *“The staff involve themselves in the continuous learning and online courses”.* Nurse in Gasabo. “*Although the clients are received without judgment, well welcoming and respectful manners, there is need for more efforts especially in counseling where some fear about coming to seek for the services*”. Social worker in Huye.

The respondents said that adolescents do not have a suitable means to provide feedback on the services being provided. This is coupled with the fact that they are less involved in suggesting ideas regarding the services that they would like to have provided to them. These made the respondents feel that the sexual and reproductive health needs of adolescents might not be met in several facilities. “*There is no transparent and confidential mechanism for adolescents to submit complaints or feedback about SRH services at the facility, but the adolescents receive results or feedback from the services delivered*”. Nurse in Huye.

“*The peer’s educator or counselors are not involved in the SRH services offered to adolescents. There is no well-organized system to receive and provide SRH care to adolescents by peers. The SRH department is not active/operative because of the lack of resources. Therefore, the people who were peer educators or peer counselors among adolescents are no longer working. Almost all of the adolescents who were in charge are students. Besides, most of the adolescents in the area surrounding this health center are the students who become available on the weekend and holidays*” Nurse in Musanze.

Respondents added that the adolescents’ needs are not being met within most of the facilities “*The needs of adolescents seeking SRH services are not met at the health center because the services are not specific and there are insufficient resources including equipment materials, medical drugs, tests and insufficient providers*” Nurse in Rubavu.

### Barriers to SRH services accessibility for adolescents

Table 5 enlists multiple factors that can impact on the accessibility and quality of services. This study also documented the obstacles that the SRH service providers perceive to be limiting seeking to access SRH services for adolescents. Major obstacles that interviewed health providers recognized were religious leaders (65.0%) and family members (68.2%) limiting adolescent’s ability to request or access the SRH services.

The in-depth interview states that the respondents’ efforts to provide adolescents with SRH services are often hindered by either religious members, community members, policies in place, and family members that limit access or seeking behaviors by adolescents.

*“The facility faces challenges including the community, family and religious leaders who influence SRH services seeking by adolescents at the facility”.* Midwife in Gasabo.

Respondents added that “C*ultural influence and religious determinants are major barriers. For example, church leaders do not accept family planning and circumcision. These barriers increase the rate of low accessibility to SRH services at the health center”.* Social worker in Rubavu

## Discussion

Our main finding was that most of the health facilities have SRH services available for provision. Rwanda seems to be doing well in availing SRH services to help adolescents access them. However, SRH services provision were designed for the general population without specialized adolescents SRH healthcare providers. Our results agree with previous studies conducted in Nigeria and Kenya that also reported that SRH services were not specifically designed for adolescent’s use [9, 18, 22]. The study data reveals that some of the services are not available in health facilities due to being underutilized, such was the case with female condoms. In addition, a predetermined health service package that is offered at a health facility as defined by the Rwanda Ministry of Health limits facilities from offering some SRH services. These results differ from findings in Nigeria, where they reported that female condoms were available in almost all health facilities included in their study [22].

Furthermore, geographical accessibility of SRH services, especially when it came to distance, was not seen to be a negative factor influencing access among adolescents’ in this study. The respondents reported that adolescents needed to walk approximately 30 min to reach a health facility. These findings may be a result of the decentralization of health systems in Rwanda for the attainment of Universal Health Coverage (UHC) [23]. Our results were different from what other studies have reported in that location is a barrier to accessing SRH services [24, 25].

Our respondents brought to light that they felt there was limited time to interact with adolescents during SRH services provision and constitutes a significant setback to SRH services accessibility. This can be explained by the fact that a shortage of the number of staff providing SRH services might be affecting the time spent providing the services. Lack of specialized Adolescents SRH courses, capacity building in Adolescents SRH services provision, full job description and organogram for SRH units’ staff might additionally explain a shortage of SRH staff due to having responsibilities within other departments. Some service providers suggested that it would be inappropriate for primary school adolescents to access SRH services irrespective of being between 10 and 19 years because they are not considered to be mature enough to make their own decisions regarding sexual health. These findings are similar to what was found by other studies that adolescents less than 12 or 14 years old cannot access SRH services without the consent of their parents, or services were rejected to be provided by healthcare providers, especially when it comes to HIV related services [24, 26].

The results demonstrate that the SRH services cost is near the standard of what was highlighted by ICPD + 5 in Beijing to promote adolescents’ access to health services [27]. This is owed to the fact that the Rwandan government has put efforts in community health insurance schemes whereby, close to all Rwandans are enrolled and insurance accepted in all public health facilities. However, a higher proportion of study respondents came from private facilities, whereby it was found that most of the services are not provided or not provided for free. This would limit adolescents who wish to access SRH services without the consent of their parents or guardians.

More than 50% of health facilities in Rwanda are affiliated to the Roman Catholic Church or other faiths who refuse to offer various SRH services including contraception and pregnancy termination. If adolescents do not have access to an insurance scheme, then they will need to pay fully for SRH services as it was observed in similar studies [14]. Our results also indicate that there is insufficient publicity to allow adolescents full access to SRH services. These results are similar to what was reported in Tanzania, whereby, SRH services for adolescents were found to be poorly publicized [28].

The current health care services provided to adolescents lag behind, especially when it concerns involving adolescents in the overall service provision process. SRH service providers did not preferentially include adolescents in serving their peers in addition to not engaging them in designing feedback mechanisms for services rendered to them or their peers. The findings align with existing knowledge that reported that SRH services provided to adolescents’ in Rwanda are not yet of the desired standard and need multi-sectorial approaches and strong coordination between and within agencies to innovatively enhance their provision [18, 29].

A safe and supportive environment for adolescents’ health agenda, as highlighted on the Action in Adolescent Health and Development by the WHO, UNICEF, and UNFPA, needs to be implemented and followed up in urban health settings that offer SRH services [20]. The SRH service providers’ views on the quality of the services for adolescents include facing several barriers mostly posed by community members, family members, and religious leaders. These barriers play a role in affecting the general quality of services. The findings are consistent with those from other low- and middle-income countries (LMICs) regarding the numerous barriers adolescents face while accessing SRH services [24, 25]. This calls for policymakers, activists, and the community to formulate methodologies to ensure that the SRH services accessibility, availability, as well as the quality of services provided are considered while designing health services provision to ensure that the “no one left behind” principle is applied to achieve Sustainable Development Goal 3 [28].

In interpreting this study, it is vital to consider the following limitations. The results could have been biased by the absence of interviewing the adolescents who directly benefit from the services. This could mean that the health care providers might have underestimated or overestimated the availability, accessibility and quality of SRH services provided to adolescents in Rwanda. Thus future studies would need to consider adolescent points of view on the subject matter.

## Conclusion

This study conducted on 159 SRH health care providers in urban settings of Rwanda has shown that SRH services are available for the general population and are not more specifically designed for adolescents. These SRH services in Rwanda seem to be fairly accessible to adolescents. Although there are private consultation rooms for providing SRH services in Rwanda, there is still an insufficient involvement of adolescents in service provision among other aspects of quality SRH services that ought to be rendered to adolescents according to WHO standards. Therefore, there is a need to improve the present quality of these services to meet adolescents’ needs in an urban setting. Adolescents’ peers would also be of great support and efforts should be made to give health care providers specific tasks on Adolescent SRH accompanied by specialized training/education on the services.

## Data Availability

The datasets used and/or analysed during the current study are available from the corresponding author on reasonable request.

## Abbreviations

SRH: Sexual and Reproductive Health
ICPD: International Conference on Population and Development
SDGs: Sustainable Development Goals
WHO: World Health Organization
SSA: Sub-Saharan Africa
HIV/AIDS: Human Immuno Virus/Acquired Immune Deficiency Syndrome
YFHS: Youth Friendly Health Services,
CMHS: College of Medicines and Health Science
SPSS: Statistical Package for the Social Sciences
UNICEF: United Nations Children’s Fund
UNFPA: United Nations Population Fund
PI: Pathfinder International
LMICs: Low and Middle Income Countries
